# The role of cerebellum in learned vocal communication in adult songbirds

**DOI:** 10.1038/s41598-024-58569-8

**Published:** 2024-04-08

**Authors:** Rebecca Radic, Kristina Lukacova, Ladislav Baciak, Vladimira Hodova, Lubica Kubikova

**Affiliations:** 1 Institute of Animal Biochemistry and Genetics, Centre of Biosciences, Slovak Academy of Sciences, 840 05 Bratislava, Slovakia; 2 Central Laboratories, Faculty of Chemical and Food Technology, Slovak University of Technology, 812 37 Bratislava, Slovakia

**Keywords:** Zebra finch, Cerebellum, Deep cerebellar nuclei (DCN), Brain, Lesions, Adult song, Fundamental frequency, Amplitude, Neuroscience, Diseases of the nervous system, Motor control

## Abstract

Injury, tumors, ischemia, and lesions in the cerebellum show the involvement of this region in human speech. The association of the cerebellum with learned birdsong has only been identified recently. Cerebellar dysfunction in young songbirds causes learning disabilities, but its role in adult songbirds has not been established. The aim of this study was to investigate the role of the deep cerebellar nuclei (DCN) in adult birdsong. We created bilateral excitotoxic lesions in the DCN of adult male zebra finches (*Taeniopygia guttata*) and recorded their songs for up to 4 months. Using magnetic resonance imaging (MRI) and immunohistochemistry, we validated the lesion efficacy. We found that the song duration significantly increased from 14 weeks post-op; the increase in duration was caused by a greater number of introductory notes as well as a greater number of syllables sung after the introductory notes. On the other hand, the motif duration decreased from 8 weeks after DCN lesions were induced, which was due to faster singing of syllables, not changes in inter-syllable interval length. DCN lesions also caused a decrease in the fundamental frequency of syllables. In summary, we showed that DCN lesions influence the temporal and acoustic features of birdsong. These results suggest that the cerebellum influences singing in adult songbirds.

## Introduction

The cerebellum has been traditionally considered to be a structure that modulates motor control^1^. However, studies involving cerebellar malformations and injuries have shown that the cerebellum is also implicated in cognitive processes and language development and performance^2–4^. Functional imaging using positron emission tomography and functional magnetic resonance imaging (fMRI) revealed the participation of the right cerebellar hemisphere in language^5–7^. Cerebellar malfunction in humans leads to ataxic dysarthria, a motor speech disorder manifested by impaired articulation and phonation^8^, changes in the loudness of speech, and the acceleration or deceleration of syllables^9^. Cerebellar lesions cause stuttering^10^, and the cerebellum has been implicated in the regulation of temporal parameters of speech^11^.

Learned speech has been studied extensively using the model of learned song in songbirds^12^. In addition to the behavioral parallels, songbirds also share homology with mammals in the brain pathways controlling vocal learning and production^13–15^. The avian pallial-basal ganglia-thalamic-pallial loop is similar to the mammalian cortical-basal ganglia-thalamic-cortical loop involved in motor learning and sensorimotor integration^16^.

The cerebellum emits outputs exclusively through the deep cerebellar nuclei (DCN), which project to various brainstem nuclei and to the cerebral cortex via the ventrolateral thalamic nuclei^1^. The effects of the cerebellum on language-controlling forebrain regions in humans can be executed via the cerebellum-thalamus-basal ganglia-cortex pathway^17,18^. Similarly, anatomical studies in songbirds revealed the indirect connection of the cerebellum with the song-related striatal part of the basal ganglia, Area X, via the dorsal thalamus^19–21^. Electrical stimulation of the DCN evoked the excitation of pallidal cells in Area X^21^. These pallidal neurons project to the thalamus^22^, which in turn sends signals to cortical-like parts of the song circuitry^14^. Additionally, neurotoxic lesions in Area X lead to structural and gene expression changes that occur not only in the dorsal thalamus but also in the cerebellum^23,24^.

The cerebellum contributes to vocal learning in young songbirds. Pidoux et al.^21^ reported that DCN lesions impair song learning in juvenile zebra finches. Young lesioned birds copied fewer song syllables from their tutor and sang the syllables faster than did the control group. DCN lesions in this study also had an impact on syllable duration in juvenile birds and affected the developmental changes in syllable duration. However, the role of the DCN in adult songbirds has not been determined.

In this study, we investigated the role of the DCN in adult songbirds. The cerebellum has been postulated to have specialized functions in language, including timing and sequencing^25–27^. Therefore, we focused on these aspects of birdsong to study the function of the DCN in adult zebra finches, but we also measured acoustic features such as fundamental frequency and amplitude.

## Materials and methods

### Experimental animals

The experiment was performed using 18 adult (7–11 months old) male zebra finches (*Taeniopygia guttata*) from the breeding colony at the Centre of Biosciences, Slovak Academy of Sciences, Institute of Animal Biochemistry and Genetics. The experiment, treatments, and procedures were approved by the State Veterinary and Food Administration of the Slovak Republic and Ethical Committee in accordance with the relevant guidelines and regulations, including the ARRIVE guidelines.

During the experiment, the males were placed in sound-attenuating boxes with a temperature of 25 ± 3 °C, humidity of 55 ± 5%, and lighting conditions of 14L:10D. Food, water, and grit were available ad libitum.

### Surgery

Birds underwent either surgery to create bilateral neurotoxic lesions in the DCN (n = 13) or a sham operation (n = 5). The birds were anesthetized using isoflurane mixed with oxygen (1.5–2.7%, 1 L/min), and the lesions were generated using 69 nL of 1% ibotenic acid solution (pH 7.6; Sigma, USA) that was pressure injected into each hemisphere via a glass capillary attached to a Nanoject II (Drummond Scientific, USA). The head angle was 50°, and the coordinates for the DCN were RC: − 2.7/− 2.85, ML: ± 1.3, and DV: − 3.4/− 3.3 from the point where the cerebellum meets the hemispheres. The micropipette was left in place for approximately 5 min after the injection to minimize possible backflow along the path of the needle. After stereotaxic surgery, the skin was sealed with acrylic glue, and the local anesthetic mesocaine (Zentiva, SK) was applied. Sham-operated birds were anesthetized, and their skulls were opened and then sealed. After 1 h of recovery under a heat lamp, the animal was returned the sound-attenuating box.

### Magnetic resonance imaging

Three days after surgery, the birds were transported to a magnetic resonance imaging (MRI) facility. MRI was used to verify proper lesion localization in the DCN. All MRI experiments were performed using a 4.7 T horizontal scanner for small animals (Agilent, Yarton, UK) equipped with a 600 mT/m gradient insert and a quadrature volume coil with an internal diameter of 25 mm (STARK contrast, Erlangen, Germany). Fifteen slices in both the coronal and sagittal orientations were obtained using a T2-weighted fast-spin echo sequence (TR/TE/NEX = 3000/80/12, ETL = 8, FOV = 30 × 30 mm^2^, matrix size = 256 × 128, slice thickness = 0.4 mm, imaging time ~ 10 min). During brain scanning, the songbirds were under isoflurane anesthesia mixed with air (induction phase: 2%, maintenance phase: 1.3–1.7%). The overall temperature inside the scanner was maintained by flowing warm air at 39 °C (SA Instruments, USA).

### Behavioral analyses

Sound Analysis Pro (SAP, 2011) software was used to record and analyze undirected songs (sung when the bird was alone). The analyzed songs were the first 10 songs sung in the morning on 2 pre-op days and post-op on days 1–3 (1d, 2d, 3d), 2 weeks (2w), and then every 2 weeks for up to 16 weeks (16w). After surgery, we observed that some of the males did not sing during the first postoperative day. Two birds started to sing on the second day, three birds on the third day, and one bird on the fourth day.

**Figure 1:**
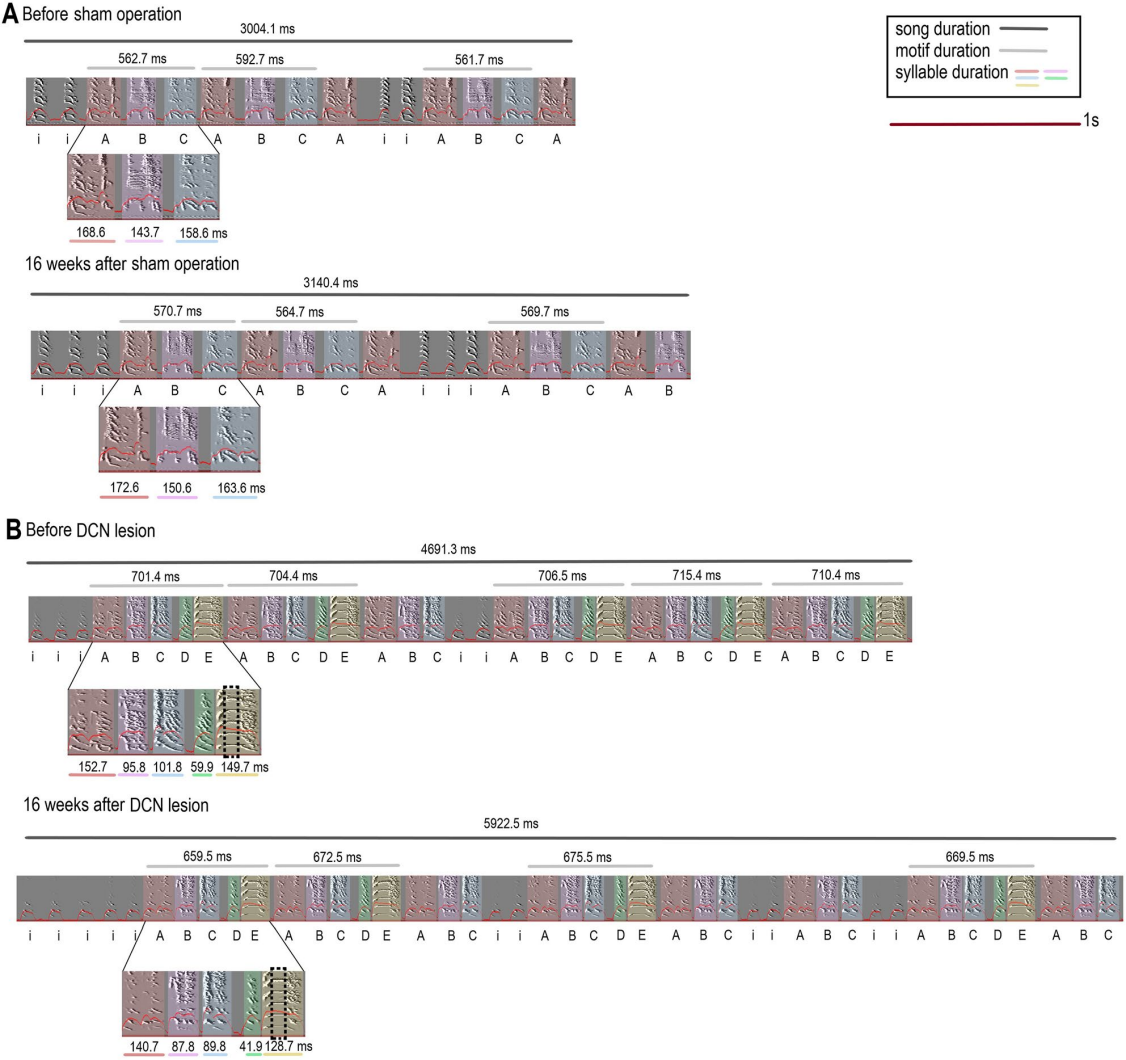
Sonograms**.** Zebra finch songs (**A**) before and after the sham operation and (**B**) before and after the DCN lesion operation. The same syllables in the song are highlighted with the same color. The black lines above the sonograms show the duration of the songs, the gray lines show the duration of the motifs consisting of the same syllables in one bird, and the color-coded lines under the sonograms show the duration of the syllables. The black dotted rectangle on syllable E in (**B**) indicates a harmonic stack where the fundamental frequency was measured.

The song of zebra finches (Fig. 1) usually starts with several introductory notes and is followed by sequences of syllables that form motifs. A motif consists of a sequence of unique syllables, and a syllable is defined as an uninterrupted sound^28^. When songs were separated by at least 2 s of silence, we considered them different songs. We measured the following parameters: the duration of the song with and without the introductory notes, total number of elements in the song (introductory notes and syllables), number of introductory notes, number of syllables in the song, number of motifs (the motif was counted only if it contained all syllables of a typical motif and not only a part of it, i.e., ABC from ABCDE), duration of the “identical motif” (consisting of the same syllables in one bird), duration of the syllables, and duration of the inter-syllable intervals. For syllables, in addition to syllable duration, we measured the syllable amplitude, pitch, mean frequency, frequency modulation, and entropy. Furthermore, we calculated the harmonic pitch for the syllables with a flat harmonic structure. This harmonic pitch is an estimate fundamental frequency (the first harmonic frequency as shown in^29^ and in Fig. 1B. The variability of each parameter was assessed using the coefficient of variation (CV) of the measurements obtained on that day.

To compare the song parameters among individuals, we normalized the data. The average of the values for each parameter on the days before surgery (pre-op1 and pre-op2) was considered 100% (average_pre-op_), and all data points were converted to percentages relative to the average_pre-op_. Each value was recalculated according to the formula a_norm_ = 100*a/average_pre-op_, where a_norm_ is the normalized value and a is the measured value.

In 2 of the DCN-lesioned birds, we observed coordination difficulties following lesioning until 2 weeks after surgery, after which the impairment disappeared. When moving, the birds lost balance. We did not record videos of this behavior. The birds sang during this time. Since their song parameters were within the range of the values obtained for other birds, we included them in the analyses.

### Tissue processing and immunohistochemistry

The birds were sacrificed 4 months after surgery with a lethal intramuscular dose of ketamine-xylazine solution (15 mg/mL ketamine and 3 mg/mL xylazine). After transcardial perfusion using 4% paraformaldehyde (PFA), the brains were postfixed in the same solution for 5 h, immersed in 20% and 30% sucrose, and frozen in Tissue Tek OCT Compound (Sakura, Japan). The brains were cut into 20 μm thick sagittal sections using a Leica 1800 cryocut (Leica, Germany), and the sections were mounted on slides.

The sections were fixed on slides for 3 min in 4% PFA and washed 3 times for 2 min in 0.1 M PBS at room temperature. Nonspecific binding was reduced by incubation in a blocking solution containing 1% bovine serum albumin (BSA, Sigma, USA) and 0.2% Triton X-100 in 0.1 M PBS for 1 h. The sections were then incubated with a rabbit anti-Fox3 antibody (Antibodies, USA; diluted 1:250 in blocking solution) for 24 h at 4 °C in a humid chamber. Afterward, the sections were washed 3 times in PBS at room temperature and incubated with goat anti-rabbit IgG conjugated with Alexa 647 (Invitrogen; diluted 1:250) for 2 h in the dark. The sections were washed 3 times in PBS for 2 min and rinsed in deionized water. The slides were coverslipped with Fluoromount G mounting medium supplemented with DAPI (Abcam).

Photomicrographs of the DCN were taken using the Leica Microsystems software LAS AF6000 connected to a Leica DM5500 fluorescence microscope and a Leica DFC 340 FX camera. Lesion size was measured in the photomicrographs with Fox3 immunostaining. Fox3 (also known as NeuN^30^) is believed to be a pan-neuronal marker. Based on the content of the Fox3^+^ neurons, the DCNs in the control animals were measured using ImageJ software (Rasband WS, NIH, Bethesda, Maryland, USA). We measured the sizes of the lateral, intermediate, and medial DCN separately and summed them to obtain the value for the whole DCN. In the lesioned birds, the unlesioned parts of the DCN were measured, and the lesion size was calculated as the difference between the average size of the DCN in the control birds and the unlesioned part in the lesioned bird and expressed as a percentage.

### Statistical analyses

The two preoperative values were compared by paired t tests and were not different for any of the measured parameters. For comparison of song parameters before and after DCN lesions at all the measured time points, one-way repeated measures analysis of variance was used, followed by Dunnett’s post hoc test for multiple comparisons with the control group or the nonparametric Holm‒Sidak test. The factor was time. To determine whether there was an association between lesion size and song parameters at 16 w, linear regressions were used.

## Results

### Lesion efficacy

**Figure 2:**
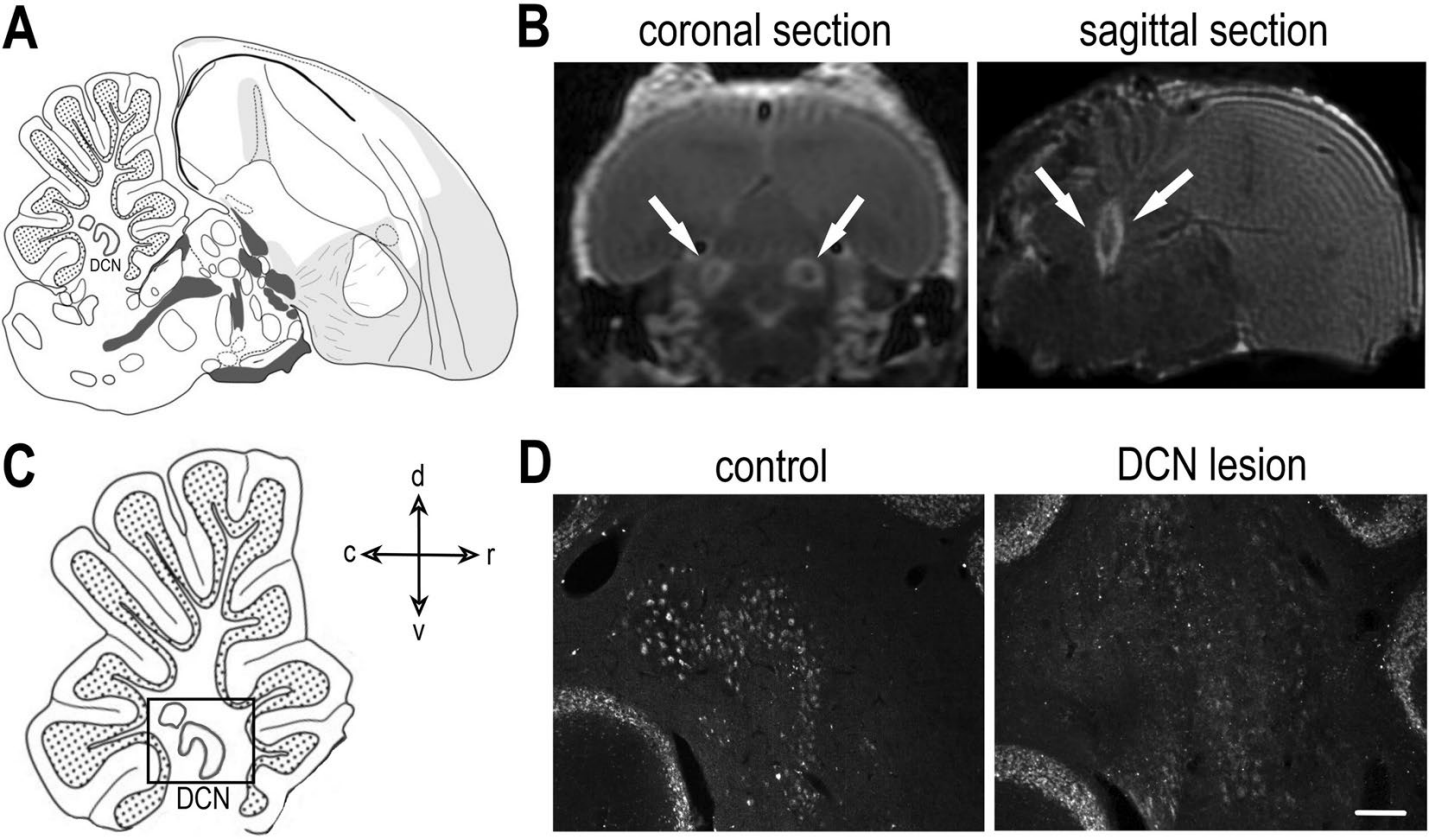
MRI brain scans and Fox3^+^ neurons in the DCN. On the left (**A**, **C**) are the slightly modified drawings captured from the zebra finch histological atlas browser (Oregon Health & Science University, Portland, OR 97239; http://www.zebrafinchatlas.org)^31^ at the position ~ 1.2 mm laterally. The cross showing the orientation applies to the drawings, MRI sagittal section and both sagittal sections in (**D**). The scans (**B**) were taken 3 days after the lesion was generated. The boundaries of the lesions on the scans have a hyperintense signal and are indicated by arrows. (**C**) A closer view of the DCN, as shown in photomicrographs of the DCN in a control and a lesioned section containing Fox3^+^ neurons (**D**). *d* dorsal, *v* ventral, *r* rostral, *c* caudal. The scale in (**D**) represents 100 μm.

Using noninvasive MRI 3 days after surgery, we evaluated the position of DCN lesions. The lesions in all experimental animals encompassed the DCN and showed a hyperintense signal on T2-weighted images (the brighter area indicated by arrows; Fig. 2A,B). Based on the neuronal Fox3^+^ staining (Fig. 2C,D), we determined that the size of the DCN lesions was 36.91 ± 5.53% (range 13.2–66.69%; 56.8 ± 9.7 for the lateral, 38.4 ± 9.9 for the intermediate, and 5.4 ± 3.4 for the medial nuclei). The lesion sizes in the two birds with initial coordination/stability difficulties were 18.4 and 43.4% (55.2 and 79.3% for lateral nuclei, 0 and 40.2% for intermediate nuclei, respectively) and the medial DCN were not lesioned. Any of the lesion size measures (overall, lateral, intermediate, or medial nuclei) did not correlate with song duration, total number of elements in the song, number of motifs, or fundamental frequency measured at 16 weeks after surgery (linear regressions: p = 0.166–0.906).

### The DCN lesions affected song duration

**Figure 3:**
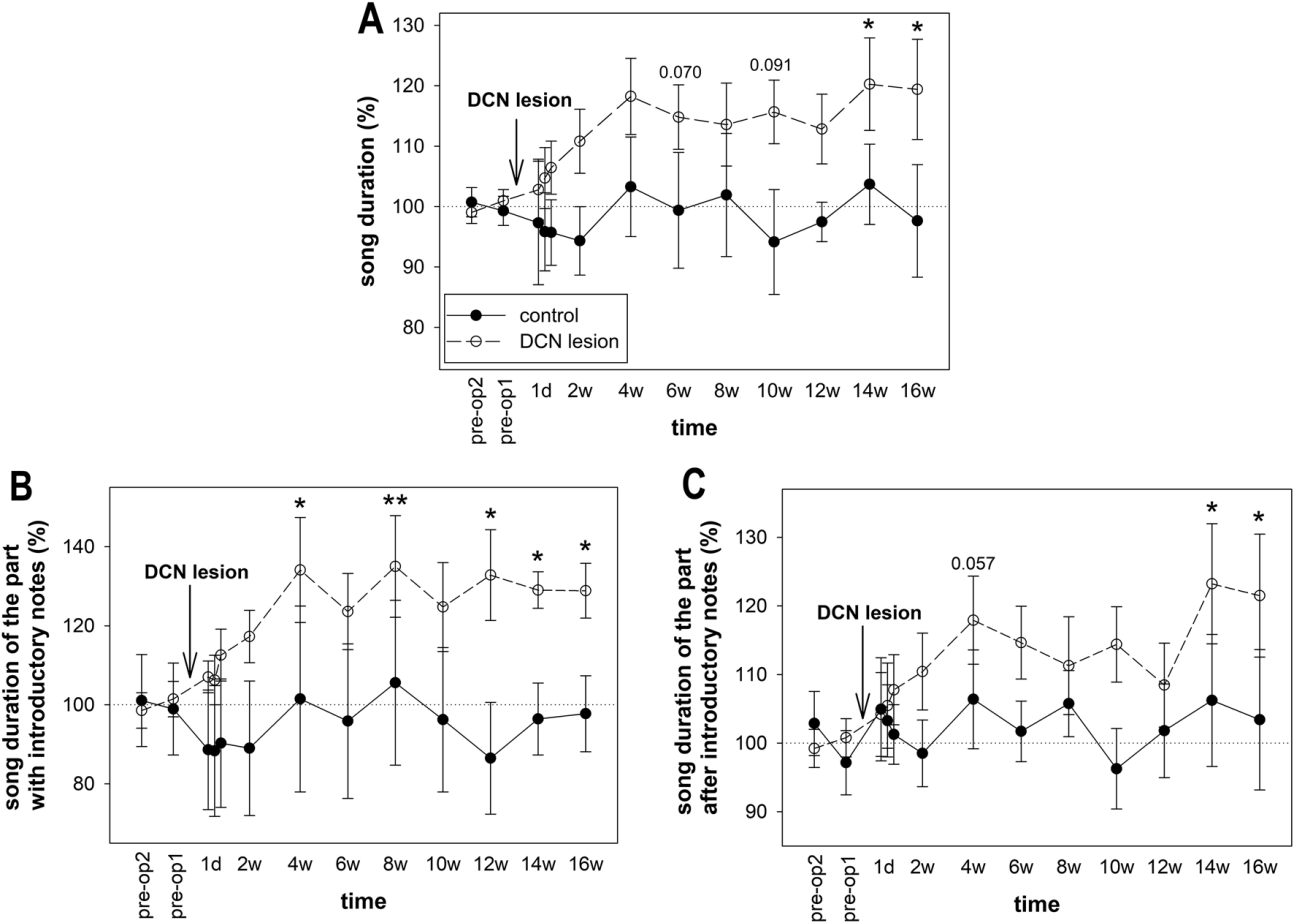
Song duration. (**A**) The duration of the entire song, (**B**) the song duration of the part with introductory notes, and (**C**) the song duration without introductory notes before and after DCN lesions. Each circle shows the mean and SEM. ANOVA followed by Dunnett’s test. *p < 0.05, **p < 0.01.

We measured the duration of complete songs. While the one-way repeated-measures ANOVA did not reveal a significant effect of time on the song duration in the control group (p = 0.976), the effect was significant in the lesioned group (p = 0.003). Dunnett’s test revealed that the birds sang longer songs at 14 w and 16 w after the DCN lesion (p = 0.046 and 0.039, respectively; Fig. 3A). The increased duration of the songs was caused by an increased duration of the part with introductory notes (ANOVA: p < 0.001; Dunnett’s test: p = 0.041 for 14 w and 0.042 for 16 w, Fig. 3B) as well as the section after the introductory notes (ANOVA: p = 0.014; Dunnett’s test: p = 0.016 for 14 w and 0.031 for 16 w; Fig. 3C).

**Figure 4:**
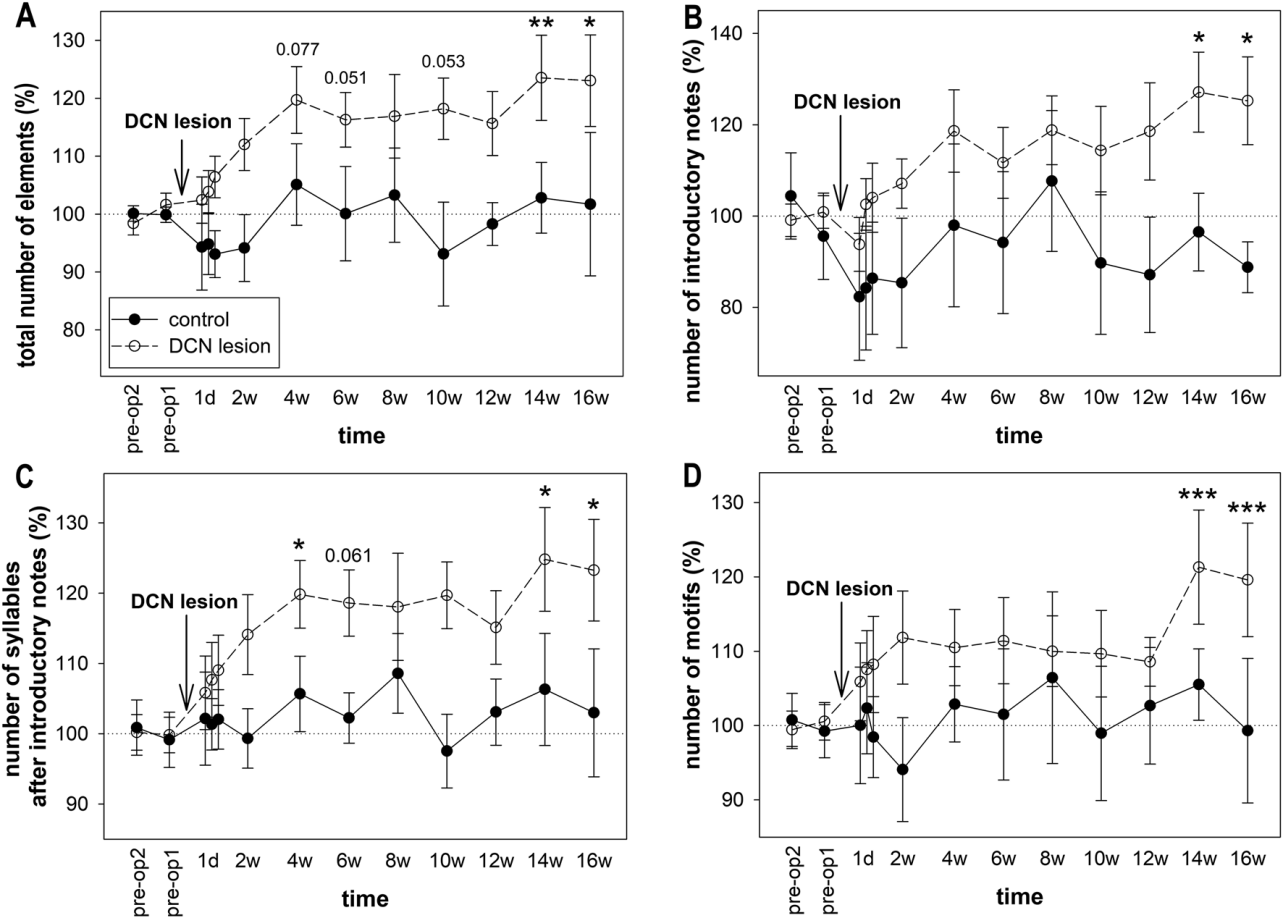
Number of syllables. (**A**) The total number of elements in the song, (**B**) the number of introductory notes, (**C**) the number of syllables in the song, and (**D**) the number of motifs. Each circle shows the mean and SEM. ANOVA followed by Dunnett’s test. *p < 0.05, **p < 0.01, **p < 0.01, ***p < 0.001.

The longer songs produced after DCN lesions could be caused by singing more song elements or by the birds singing the same number of elements more slowly. Therefore, we next quantified the number of elements (introductory notes and syllables) in the songs. We found that the time since the lesion affected the total number of elements in the song (ANOVA: p < 0.001), and the song consisted of significantly more elements at 14 weeks and 16 weeks after the DCN lesion (Dunnett’s test p = 0.008 and 0.014, respectively; Fig. 4A). The time since the sham surgery did not influence the number of syllables in the whole song in the control birds (ANOVA: p = 0.764). More detailed analyses revealed that both the number of introductory notes (ANOVA: p = 0.003; Dunnett’s test: p = 0.026 for 14 weeks and 0.046 for 16 weeks; Fig. 4B) and the number of syllables following the introductory notes (ANOVA: p = 0.002; Dunnett’s test: p = 0.036 for 14 weeks and 0.039 for 16 weeks; Fig. 4C) were increased in birds with DCN lesions. These syllables formed typical motifs (e.g., ABCDE) or motifs with fewer syllables than usual (e.g., ABC) or were situated between motifs (e.g., ABCDE × ABCDE). We calculated the number of typical motifs and found that it increased after DCN lesion (ANOVA: p < 0.001; Dunnett’s test: p < 0.001 for 14 weeks and p < 0.001 for 16 weeks; Fig. 4D).

We also assessed trial-to-trial variability (measured by CV) for song duration, duration of the part with/after introductory notes, total number of elements, number of introductory notes, number of syllables, and number of motifs. ANOVA revealed no effect of time after operation on the CV of these parameters in control or lesioned birds.

In summary, we found that DCN lesions caused birds to sing longer songs that contained more introductory notes as well as more song syllables, resulting in the formation of more motifs. Next, we wanted to distinguish whether the birds only sang more of the song elements or whether the tempo of the song was also changed.

### DCN lesions affected the song tempo

**Figure 5:**
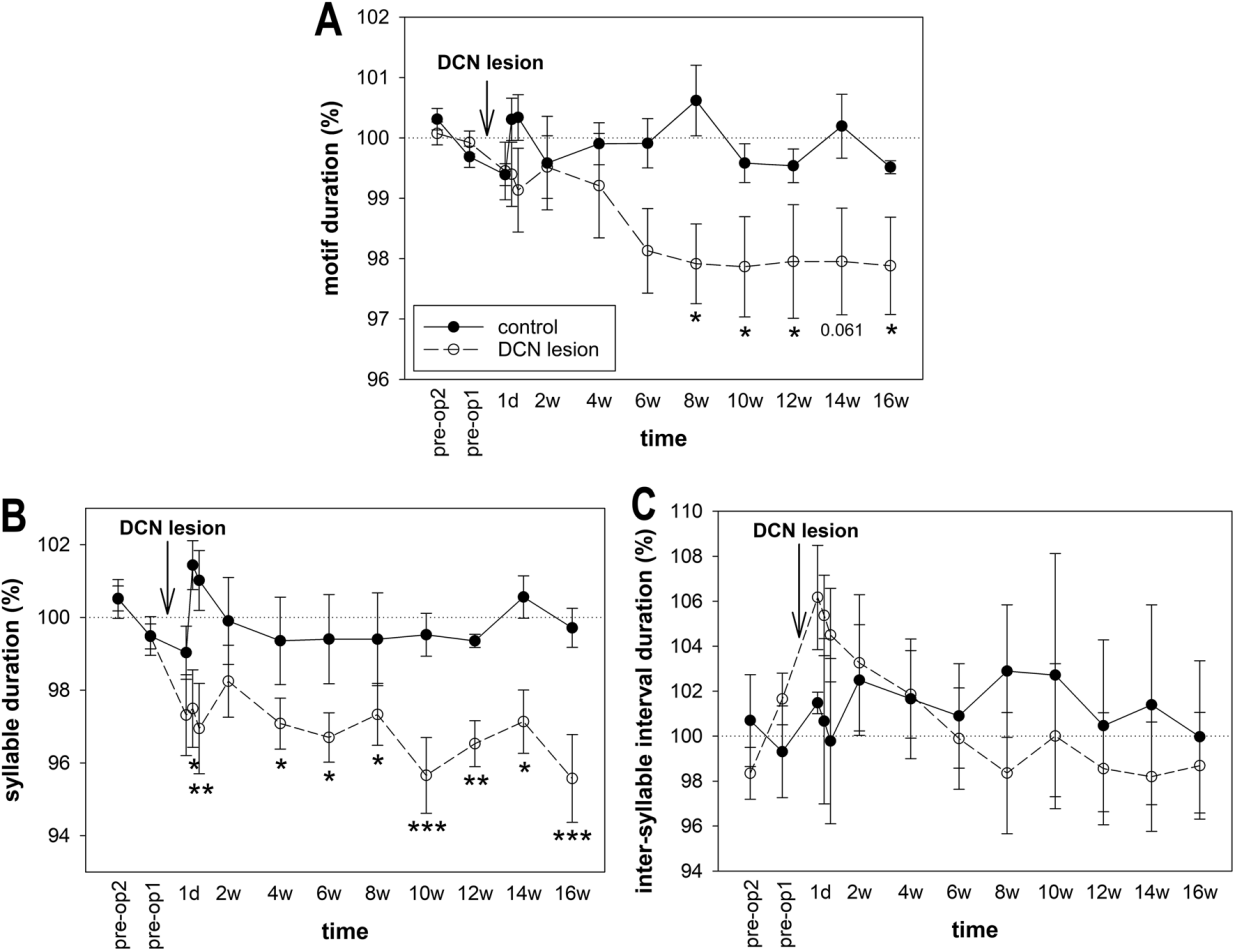
Song tempo. (**A**) Motif duration, (**B**) syllable duration, and (**C**) inter-syllable interval duration. Each circle shows the mean and SEM. ANOVA followed by Dunnett’s test. *p < 0.05, **p < 0.01, ***p < 0.001.

We measured the duration of motifs containing identical syllable sequences in the same bird and found that it was not affected by time in the control birds (p = 0.413). However, the motif duration in the lesioned birds became significantly shorter from 8 weeks onward (ANOVA: p < 0.001; Dunnett’s test: p = 0.010–0.026 for 8 weeks–16 weeks; Fig. 5A), except at 14 weeks, when there was a trend toward shorter motif duration (p = 0.061). Further analyses revealed that the motifs were shorter due to shorter syllables that decreased in length immediately after the DCN lesion (ANOVA: p < 0.001; Dunnett’s test: p = 0.08 for 2 days, p < 0.001–0.047 for 3 days–16 weeks, except for p = 0.26 at 2 weeks; Fig. 5B). The inter-syllable intervals within the song motifs did not change the duration (ANOVA: p = 0.999; Fig. 5C). The length of the syllables before surgery ranged from 88.4 ± 9.5 ms to 219.1 ± 17.4 ms, and the length of the inter-syllable intervals ranged from 27.1 ± 2.9 ms to 64.5 ± 4.1 ms. There was no effect of the time after operation on the variability (CV) of motif duration, syllable duration, or inter-syllable interval in the control or lesioned birds.

### DCN lesions affected the acoustic features of syllables

**Figure 6:**
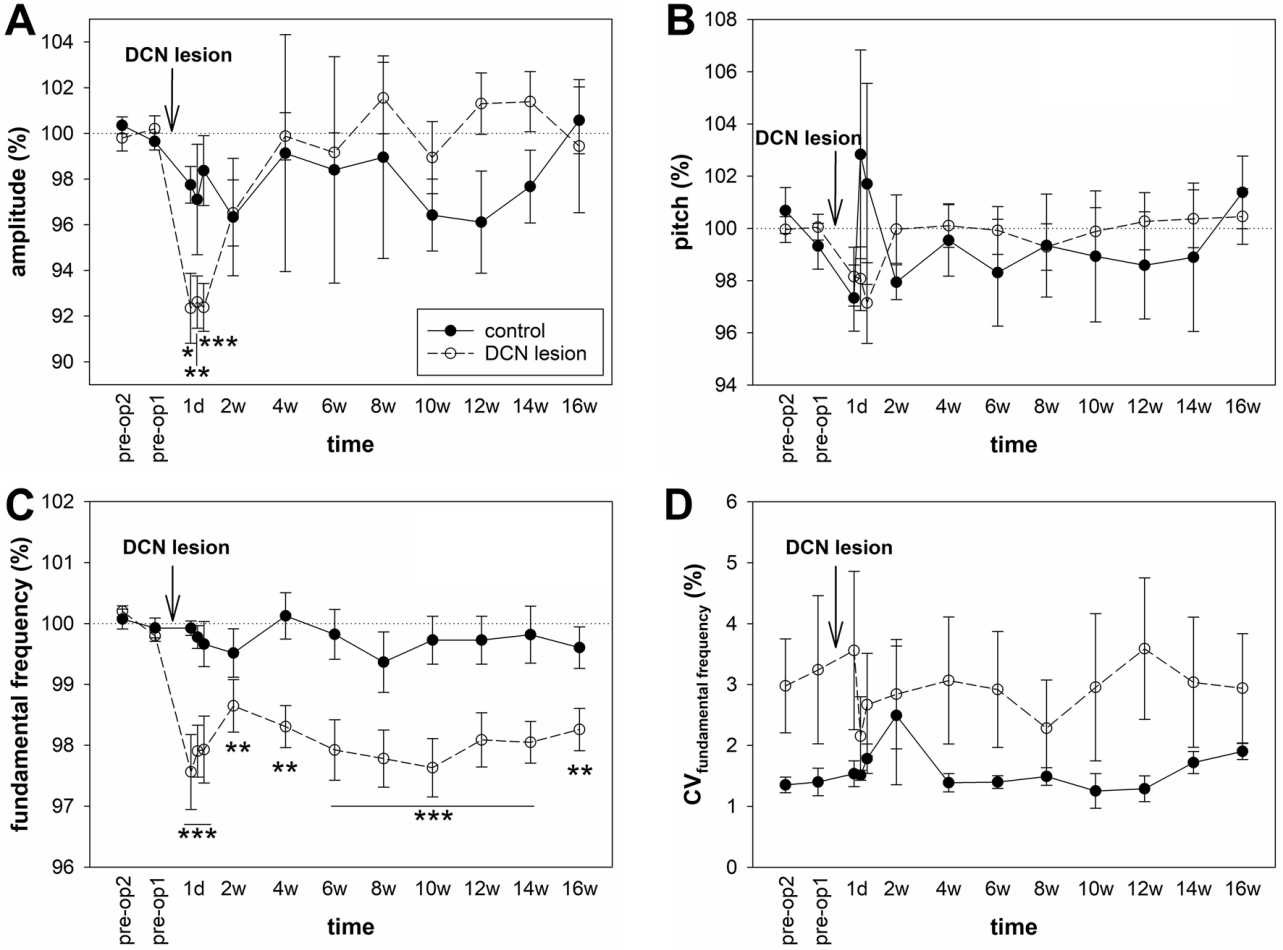
Acoustic parameters. (**A**) Amplitude and (**B**) pitch of all syllables, (**C**) fundamental frequency of harmonic syllables, and (**D**) CV of the fundamental frequency of the harmonic syllables. Each circle shows the mean and SEM. ANOVA followed by Dunnett’s test. *p < 0.05, **p < 0.01, ***p < 0.001. The stars below the lines in (**C**) represent the statistical significance for each of the selected time points.

Next, we assessed the acoustic features of the syllables. We found that the time after the lesion affected the amplitude of syllables (ANOVA: p < 0.001) but not the trial-to-trial variability (measured by CV). The amplitude was significantly lower during the first 3 days after lesion (Dunnett’s test: p = 0.017 for 1 day, p = 0.002 for 2 days, p < 0.001 for 3 days), after which the amplitude increased to the pre-operative levels (Fig. 6A). Time did not affect the other measured parameters of syllables, mean frequency, frequency modulation, entropy, pitch (Fig. 6B), or  their CV in lesioned or control birds.

Since DCN is linked to the striatal vocal region Area X^21^ and lesions in Area X result in a decreased fundamental frequency within harmonic syllables^32^, we measured the fundamental frequency of the syllables that contained the harmonic part (n = 8 in control birds, n = 25 in lesioned birds). We found that the time after the lesion affected the fundamental frequency in lesioned birds (ANOVA: p < 0.001), and the frequency decreased immediately after the DCN lesion (Dunnett’s test: p = 0.008–p < 0.001; Fig. 6C). The time after the sham operation did not affect the fundamental frequency in the control birds. Time did not affect the variability (CV) of the fundamental frequency in the control or lesioned birds (Fig. 6D).

## Discussion

This study is the first to show the role of the cerebellum in learned vocalization in adult male songbirds. The cerebellum has been hypothesized to affect language timing and sequencing^25–27^ and variability in motor tasks^33,34^. Here, we focused on these aspects of birdsong and investigated the hypothesis that cerebellar damage in the DCN can influence learned birdsong in adults. We found that neurotoxic damage to the DCN leads to temporal changes, such as the length and tempo of the songs, as well as to acoustic changes, including the fundamental frequency and amplitude.

The cerebellum is involved in muscle coordination, articulation, and rhythm of speech. Cerebellar circuits in the supplemental motor area, Broca’s area, basal ganglia, and thalamus are linked to the planning, initiation, and coordination of speech^35,36^. The cerebellum is one of the key brain structures involved in time perception^35,37^. We found that male zebra finches in our study sang longer songs after bilateral DCN lesions occurred, while the length of the songs did not change for the sham-operated birds. Longer songs could be caused by singing more song elements (introductory notes, syllables), singing the same number of elements more slowly, or a combination of the changed number of elements and tempo. We found that longer songs contained more introductory notes as well as syllables. The length of the song was also affected by manipulating the temperature in HVC, where cooling HVC decreased the number of syllables, while warming HVC had the opposite effect^38^. The number of vocal outputs per time period in that study correlated positively with HVC temperature, but changing temperature in the downstream robust nucleus of the arcopallium (RA) did not affect the length of the songs.

In addition to the fact that the birds with DCN lesions sang longer songs, the tempo also changed, and the birds sang the song motifs faster. More detailed analyses revealed that the birds sang the syllables faster, but they did not change the length of the inter-syllable intervals. In a previous study, DCN lesions in young zebra finches caused similar changes in syllable duration within a week after surgery^21^. On the other hand, cerebellar dysfunctions in humans lead to slowed speech tempo^39–41^, and cerebellar ataxia can be considered a temporal control interruption^42^.

The cerebellum has been studied for its involvement in the processing of temporal information of various lengths^43,44^. In humans, cerebellar lesions decrease discrimination at long (4 s) and short (400 ms) time intervals^45^ and impair the production of intervals longer than 10 s^46^. We found that DCN lesions in zebra finches affected the length of syllables, ranging from 88.4 to 219.3 ms in our study, which corresponds to the length of acoustic segments of speech^47^. Similarly, people with cerebellar dysfunction are unable to correctly recognize time intervals of 10–150 ms^48^. On the other hand, zebra finches after DCN lesion in our experiment did not change the length of the inter-syllable intervals, ranging from 27.1 to 64.5 ms. Zebra finches, however, are able to change even these short intervals, as shown by the extended inter-syllable intervals 1–28 days following the occurrence of Area X lesions^49^.

Dyslexia is linked to a defect in the cerebellum and involves difficulties with writing or typing, but many individuals with dyslexia also have auditory problems. Distinguishing letter sounds (phonemes) relies on detecting variations in sound frequency and amplitude^50^. The fundamental frequency represents the pitch of a sound composed of harmonically related frequencies. Zebra finch song, however, consists of syllables with and without harmonic stacks. Here, we measured the pitch of all syllables and, specifically, the pitch (fundamental frequency) of the harmonic part of the syllables. Although the pitch of the syllables generally did not change after the cerebellar lesions developed, the fundamental frequency of the harmonic part of the syllables decreased. In addition, we detected decreased amplitudes after DCN lesion. While the fundamental frequency decreased until the end of the experiment at 16 weeks after surgery, the effect on amplitude was immediate and transient. Pidoux et al.^21^ did not observe an effect of DCN lesions on the fundamental frequency or amplitude of syllables in adult or juvenile zebra finches. However, the nuclei within the pallial-basal ganglia-thalamic-pallial loop, Area X and LMAN, have been shown to drive changes in the fundamental frequency of syllables^32,51,52^.

Inhibition in the lateral cerebellum leads to increased variability in motor actions^33,34^. Lesions in the LMAN and Area X affect variability in the fundamental frequency of harmonic syllables^29,51^, but lesions within the lateral DCN do not have a similar effect on CV of the fundamental frequency in juvenile or adult zebra finches^21^. Similarly, in our study, we did not find any effect of DCN lesions on the variability (CV) of the fundamental frequency or any of the temporal or acoustic features that we measured.

Cerebellar mutism is another speech disorder that is clinically manifested mostly by a temporary loss of speech output. The impairment occurs in the case of tumors in the cerebellum^53^ or as a post-operative syndrome^27,54^. In this study, we also observed that some of the males 1–3 days post-op did not sing. It is possible that as in humans, lesions in DCN in zebra finches might cause a temporary mutism.

Cerebellar functions in humans are executed via connectivity with other brain centers, such as the thalamus, supplementary motor area and cortical-associative areas, providing anatomical substrates for speech control^4,55^. The cerebellum is linked to the basal ganglia, forms a cerebello-thalamo-cortical pathway, and plays a relevant role in speech acquisition in childhood and mature speech production^55,56^. Similarly, in songbirds, the projection from the cerebellum to the striatal nucleus Area X via the dorsal thalamic zone was identified^19–21^. The DCN send excitatory projections to Area X, and this activity modulation of the basal ganglia is then propagated via the thalamus to the cortical lateral magnocellular nucleus of the anterior nidopallium (LMAN), which sends a projection to the premotor nucleus RA^21,57^. Alternatively, the DCN could affect song production via the connection of the dorsal thalamus to the cortical medial magnocellular nucleus of the anterior nidopallium (MMAN) to the motor pathway nucleus HVC (proper name^58^), which projects to the RA^59–61^.

The connection between the cerebellum and the motor cortex is known to be important for motor coordination and motor control^1,62^. To avoid potential impairment of these cerebellar functions, we limited the size of our lesions. Despite this, we observed two birds with coordination difficulties that disappeared within 2 weeks after surgery. The motor problems were not caused by excessive lesions, as the lesion sizes of these birds fell within the range of those of the other birds. Large lesions in the medial DCN can affect many critical functions and cause seizures, hypotonia, dysmetria, and tremors^63,64^. However, the lesions in the birds in our study were located more often in the lateral and intermediate parts of the DCN, and the two birds with motor problems did not have lesions within the medial DCN. They sang during this time, and the song features that were quantified were within the ranges of those measured in the other lesioned birds. Therefore, we included the data in the analyses.

Injury induced by the neurotoxin ibotenic acid in striatal Area X of zebra finches induces neurogenesis, and neurons start to recover within the first month after lesion induction^23,49,65^. Granule cells in the early postnatal rodent cerebellum can be recovered after depletion^66^, and there is some controversial evidence about cerebellar neurogenesis even in adults^67^. Although neurons within the DCN have not been associated with adult neurogenesis, we employed MRI to verify the lesion location soon after surgery.

## Conclusions

For the first time, our study demonstrated the significant influence of the cerebellum on the songs of adult songbirds. We observed significant modifications at the temporal and acoustic levels of the songs and changes in the number of syllables and motifs. Similar to humans, the cerebellum can affect learned vocalization, and even smaller injuries in the cerebellum might impair the adult production of birdsong.

## Data Availability

Data will be made available on request from the corresponding author.
